# Dynamics of Neutrophils-to-Lymphocyte Ratio Predict Outcomes of PD-1/PD-L1 Blockade

**DOI:** 10.1155/2017/1506824

**Published:** 2017-11-28

**Authors:** Michele Moschetta, Mario Uccello, Benjamin Kasenda, Gabriel Mak, Anissa McClelland, Stergios Boussios, Martin Forster, Hendrik-Tobias Arkenau

**Affiliations:** ^1^Drug Development Unit, Sarah Cannon Research Institute, London, UK; ^2^University Hospital of Basel, Basel, Switzerland; ^3^The University College London Cancer Institute, London, UK; ^4^Department of Medical Oncology, University of Ioannina, Ioannina, Greece

## Abstract

**Introduction:**

Baseline neutrophil-to-lymphocyte ratio (NLR) has been repeatedly reported as a significant prognostic factor in advanced cancer patients. We explored whether changes in NLR may predict outcome of advanced cancer patients enrolled into phase 1 trials and treated with PD-1/PD-L1 inhibitors.

**Patients and Methods:**

Advanced cancer patients enrolled into phase 1 trials between September 2013 and May 2016 and treated with anti-PD-1/PD-L1 agents were included in this retrospective study. NLR was calculated at baseline and after 2 cycles of treatment. Royal Marsden Hospital (RMH) prognostic score and Eastern Cooperative Group (ECOG) performance status (PS) were determined at baseline. Kaplan-Meier estimation and Cox regression analyses were used to assess the impact of NLR dynamics on PFS.

**Results:**

Among the 55 patients eligible, 26 (47%) were treated with anti-PD-L1 monotherapy, 22 (40%) received single agent anti-PD-1, and 7 (13%) were given a tyrosine kinase inhibitor (TKI) plus a PD-1 inhibitor. Neither ECOG PS nor RMH prognostic score was significantly associated with PFS in our cohort, whereas changes in NLR significantly impacted on PFS.

**Conclusion:**

Changes in the NLR may be a useful predicting factor in advanced cancer patients treated with anti-PD-1/PD-L1 agents. Further prospective trials are needed to verify these findings.

## 1. Introduction

Immune checkpoint inhibitors have emerged as potent and effective treatments for various types of haematological and solid malignancies [1]. In particular, blockade of the PD-1/PD-L1 axis can result in dramatic and sustained tumour regression in multiple cancer types [2, 3]. Under normal circumstances, this pathway is necessary to maintain immune homeostasis [4]. When PD-L1 binds to PD-1, an inhibitory signal is transmitted into the T-cell, protecting normal cells from collateral damage. Nevertheless, upregulation of PD-L1 may allow cancer cells to evade immune surveillance [3]. Considering the costs and potential side effects of novel anti-PD-1/PD-L1 agents, it is of vital importance to identify reliable biomarkers to select the most suitable patients for these drugs while sparing nonresponders from toxicity.

PD-L1 expression as determined by immunohistochemistry is considered the most useful biomarker in predicting outcomes of PD-1/PD-L1 blockade [4]. Several studies have investigated the role of PD-L1 expression in tumour and stromal cells as a potential biomarker of response, but the results were somewhat contradictory [4, 5]. Indeed, several factors can limit the reliability of this biomarker, including the use of different monoclonal antibodies for detection of PD-L1, variable procedures for biopsy collection and storage, lack of defined thresholds to describe PD-L1 expression in samples, and intratumour heterogeneity in PD-L1 expression [5] The presence of microsatellite instability, tumour mutational load, tumour-infiltrating lymphocytes (TILs), myeloid-derived suppressor cells (MDSCs), indoleamine 2,3-dioxygenase, regulatory T cells, and immune specific signatures have been also investigated with promising results [6–8]. Despite the aforementioned methods, there is still a lack of a simple, effective, and definitive biomarker of response to immune checkpoint inhibitors.

Increased neutrophil-to-lymphocyte ratio (NLR) has been reported as an independent poor prognostic indicator in several malignancies and its normalisation following treatment has been found to predict survival in cancer patients considered for early phase clinical trials [9]. Here, we investigated the usefulness of NRL changes in predicting progression-free survival (PFS) in patients undergoing treatment with PD-1/PD-L1 inhibitors within phase 1 clinical trials.

## 2. Patients and Methods

Data of metastatic cancer patients enrolled in phase 1 trials between September 2013 and May 2016 in our institution were retrospectively reviewed. Patients treated with PD-1/PD-L1 checkpoint-directed therapy were eligible. All the subjects had a histologically confirmed diagnosis of metastatic solid cancer and were intended to receive treatment with an anti-PD-1/PD-L1 agent given as monotherapy or in combination with a tyrosine kinase inhibitor (TKI). Baseline parameters, tumour characteristics, and treatment data were all reviewed and anonymously collected for this study. All the subjects met the standard inclusion criteria for phase 1 trials: Eastern Cooperative Group (ECOG) performance status (PS) 0 or 1; measurable disease based on Response Evaluation Criteria in Solid Tumour (RECIST); adequate bone marrow, liver, and kidney function; life expectancy of at least 3 months. Baseline characteristics recorded in the eligible population included demographic variables, tumour type, anticancer treatment (anti-PD-1 versus anti-PD-L1 versus anti-PD-L1 plus TKI), number of previous lines for metastatic disease, Royal Marsden Hospital (RMH) prognostic score [10], white blood cell (WBC) level, absolute neutrophil count (ANC), absolute lymphocyte count (ALC), and neutrophil-to-lymphocyte ratio (NLR). The RMH prognostic score (range 0–3) was calculated at baseline, taking into account albumin level, lactate dehydrogenase (LDH) level, and number of metastatic sites [10]. The NLR was calculated using the standard formula: NLR = ANC/ALC. NLR was calculated at baseline (cycle 1 day 1), and after 6 weeks (2 cycles) of treatment. Patients were treated until disease progression, death, or unacceptable toxicity. We considered PFS as our main outcome, which was defined as the time from treatment start until progression or death, whichever occurred first.

To investigate the dynamics in NLR between baseline and after 2 cycles of anti-PD-1/PD-L1 therapy, we used a landmark approach by excluding patients who were not able to receive at least 2 cycles of treatment to avoid guarantee time bias. We used multivariate Cox regression analyses with the relative NLR difference as independent and PFS as the dependent variable. To adjust for possible confounding, we introduced the RMH score into the model and additionally added a random effect for tumour entity, in order to account for possible heterogeneity between tumour types. We calculated univariate and multivariate hazard ratios (HR) with accompanied 95% confidence intervals (CI); however, the multivariable analysis is considered as main analysis. To visualize the prognostic effect of the NLR difference, we created Kaplan-Meier plots. All *p* values are exploratory in nature with a conventional level of significance at 0.05. All analyses were done using the statistical software R (https://www.r-project.org/) and STATA (version 14).

## 3. Results

A total of 67 potentially eligible patients were identified. Of those, 12 subjects received less than 2 cycles and were therefore excluded from the analysis. The characteristics of the included 55 patients are summarised in Table 1. Median age of patients included was 61 years (40 to 80 years). The most represented tumour type was non-small cell lung cancer (NSCLC) with 18 (33%) subjects, followed by upper gastrointestinal (*n* = 10; 18%), bladder (*n* = 8; 15%), and breast (*n* = 7; 13%) carcinomas. Median number of previous lines of treatment for metastatic disease was 1 (range 1–6) while baseline median number of metastatic sites of disease was 2 (range 1–4). RMH prognostic score at baseline was 0 in 31 (56%) subjects and 1 or higher in 24 (64%) subjects. Proportion of patients with ECOG PS 0 or 1 was 36 (65%) and 19 (35%), respectively. In total, 26 (47%) of the patients were treated with anti-PD-L1 monotherapy, 22 (40%) received single agent anti-PD-1, and 7 (13%) were given a TKI in combination with a PD-1 inhibitor.

**Figure 1:**
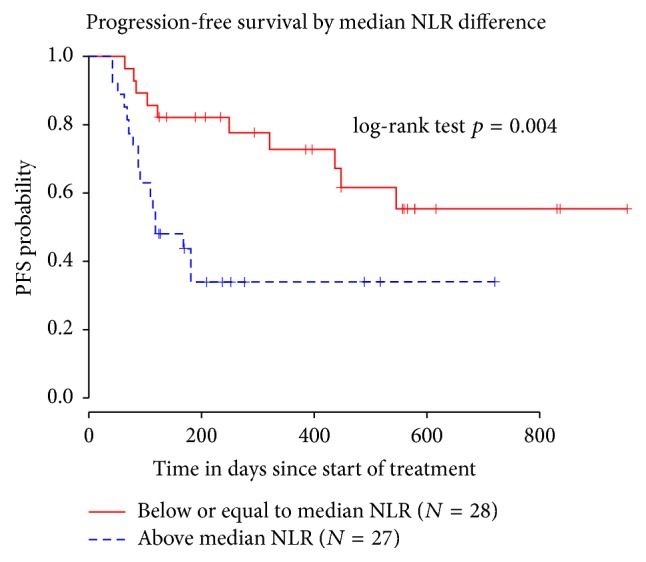
Progression-free survival (PFS) stratified by median differences in neutrophil-to-lymphocyte ratio (NLR) between baseline and after 2 doses of treatment with an anti-PD-1/PD-L1 inhibitor, showing longer PFS in patients with a reduction of NLR compared to the median baseline NLR.

On univariate analysis, baseline NLR, treatment modality, RMH score, ECOG PS, and number of metastatic sites did not have significant impact on PFS. Baseline median NLR was 3.4 in the overall population. Patient population was divided into 2 distinct groups based on decrease (Group A) or increase (Group B) of NLR in comparison with median NLR after treatment with a PD-1/PD-L1 inhibitor. No substantial differences in distribution were observed between these 2 groups in terms of age, sex, type of treatment, ECOG PS, and RMH prognostic score (Table 2). Increased NLR after 2 cycles of anti-PD-1/PD-L1 therapy had a negative effect on PFS (HR 1.14, 95% CI 1.06–1.23, *p*  =  0.004), (Figure 1). This effect was also observed in our multivariate analysis, where increased NLR was associated with decreased PFS after adjusting for RMH prognostic score (HR 1.03, 95% CI 1.01–1.04, *p* < 0.001). Baseline median ANC level was significantly higher in Group A than in Group B (*p* = 0.029). In Group A, a reduction in median ANC was shown after 2 cycles of treatment when compared with baseline ANC level, whereas this was not observed in Group B. After 2 cycles of treatment with anti-PD-1/PD-L1 agent, median ANC was significantly higher in Group B when compared to Group A (*p* = 0.014). Median ALC did not change significantly after treatment (*p* = 0.222) and no significant differences were shown between baseline and posttreatment values in both Group A and Group B (*p* = 0.24) (Table 2).

## 4. Discussion

Molecular selection in patients undergoing treatment with immune checkpoint inhibitors is an urgent unmet medical need. Ongoing approval of several anti-PD-1/PD-L1 agents and the emergence of safety concerns from immune-related adverse events also highlight the need for biomolecular stratification. Several biomarkers have been investigated, some of which have shown potential usefulness in predicting the activity of these agents. So far, PD-L1 expression in tumour cells remains the most reliable but many technical limitations have been associated with this biomarker [4, 5]. Furthermore, individuals with negative PD-L1 expression can still respond to PD-1/PD-L1 blockade, further questioning the value of PD-L1 expression as a universal biomarker [11]. Therefore, alternative markers of response need to be identified.

Clinicopathologic factors have been extensively investigated in several tumour types and anticancer therapies. Among them, ECOG PS has been repeatedly reported as a strong predictor of survival in multiple settings. The RMH prognostic score was electively implemented in advanced cancer patients enrolled in phase 1 studies [10]. Unexpectedly, neither ECOG PS nor RMH prognostic score were significantly associated with PFS in our cohort. Our results are not consistent with previous studies showing significant prognostic significance of RMH score and ECOG PS in phase 1 trial patients [10, 12, 13].

Baseline NLR has been reported to predict overall survival in cancer patients undergoing both conventional chemotherapy and targeted treatments, including immune checkpoint inhibitors. In previous reports, a correlation between baseline NLR and survival was shown in kidney cancer and NSCLC treated with IL-2 and nivolumab, respectively [14, 15]. Conversely, in our group of patients, baseline NLR was not found to correlate with PFS, although this result may have been determined by the limited sample size. In our study, we retrospectively analysed phase 1 trial cancer patients with advanced disease who had received at least 2 cycles of treatment with anti-PD-1/PD-L1 agent, to assess the significance of NLR as an independent biomarker in predicting clinical benefit in terms of PFS. Interestingly, a decrease in NLR after 2 cycles of treatment with PD-1/PD-L1 blockade was associated with longer PFS in our cohort. Accordingly, changes in NLR had shown to predict better outcomes in cancer patients undergoing conventional chemotherapy or targeted treatment but had been never investigated in subjects receiving immune checkpoint inhibitors [16–18].

Another important finding in this study was the observation that negative or positive changes in NLR were driven by a decrease in ANC and not by changes in ALC as one would have expected. Though retrospective in nature, our findings on ANC may be interpreted as hypothesis-generating. Despite the fact the T lymphocyte activity is the main target of PD-1/PD-L1 blockade, our results may suggest an important interaction between the neutrophils and tumour microenvironment. We may also speculate that the systemic effect of anti-PD-1/PD-L1 agents involves a crucial effect on circulating myeloid populations included in the ANC as measured by automated full blood cell count analyser. Preclinical evidence shows that MDSCs can impair the efficacy of immunotherapy [19]. Under physiological conditions, there is a low level of MDSCs in the bloodstream while these populations rapidly expand during immunological responses to infections, inflammation, and cancer [20]. MDSCs can adopt multiple mechanisms to induce immunosuppression, including production of arginase 1 and inducible nitric oxide synthase, leading to T-cell inhibition [19, 20]. MDSCs are also known to enhance cancer cell proliferation, confer resistance to anticancer therapies, and promote angiogenesis and metastasis [20]. Concomitant targeting of MDSCs may therefore increase the antitumour activity of PD-1/PD-L1 inhibitors in nonresponders. Moreover, a decreased mobilisation of MDSCs from the bone marrow may represent a systemic effect of anti-PD-1/PD-L1 treatment that needs to be better investigated in preclinical studies. A subsequent decrease of tumour-infiltrating MDSCs may then unleash antitumour activity of TILs and ultimately contribute to the therapeutic effect of anti-PD-1/PD-L1 agents.

Our study has several biases, including its retrospective nature, limited sample size, heterogeneous tumour types, and the choice of PFS as endpoint instead of overall survival. Nevertheless, we showed that NLR, a simple haematological parameter easily obtainable in daily clinical practice, may be used to predict clinical benefit from PD-1/PD-L1 inhibitors. These results are in line with common clinical experience with these agents, where a rapid clinical benefit can be observed despite unusual initial patterns of imaging response. Further studies conducted in larger prospective cohorts of patients undergoing treatment with immune checkpoint inhibitors are needed to confirm the predictive role of NLR in this setting.

## Figures and Tables

**Table 1:** Patients' characteristics at baseline. NCSLC = non-small cell lung cancer; ECOG PS = Eastern Cooperative Oncology Group performance status; GI = gastrointestinal; TKI = tyrosine kinase inhibitor; RMH = Royal Marsden Hospital.

Characteristic	*n* (%)
Sex	
Male	19 (35)
Female	36 (65)
ECOG PS	
0	36 (65)
1	19 (35)
>1	—
Tumour type	
NSCLC	18 (33)
Upper GI cancer	11 (20)
Bladder cancer	8 (15)
Renal cell carcinoma	8 (15)
Breast cancer	7 (13)
Colorectal cancer	2 (4)
Ovarian cancer	1 (2)
Therapy	
Anti-PD-1	22 (40)
Anti-PD-L1	26 (47)
Anti-PD-L1 plus TKI	7 (13)
RMH prognostic score	
0	31 (56)
1	19 (35)
2	3 (5)
3	2 (4)
	Median (range)
Age	61 (40–80)
Number of metastatic sites	2 (1–4)
Number of previous treatment lines	1 (1–6)

**Table 2:** Distribution of patient population in two groups. Group A: neutrophil-to-lymphocyte ratio (NLR) after 2 cycles ≤ median baseline NLR. Group B: NLR after 2 cycles > median baseline NLR. ECOG PS = Eastern Cooperative Oncology Group performance status; RMH = Royal Marsden Hospital; ANC = absolute neutrophil count; ALC = absolute lymphocyte count; SD = standard deviation; IQR = interquartile range; NLR = neutrophil-to-lymphocyte ratio.

Characteristic	Group A (*n* = 28)	Group B (*n* = 27)
Sex *n* (%)		
Female	11 (39)	8 (30)
Male	17 (61)	19 (70)
ECOG PS *n* (%)		
0	16 (43)	20 (74)
1	12 (57)	7 (26)
RMH prognostic score *n* (%)		
0-1	25 (89)	25 (93)
2-3	3 (11)	2 (7)
Intervention *n* (%)		
Anti-PD-1	14 (50)	8 (30)
Anti-PD-L1	9 (32)	17 (63)
Anti-PD-1 plus TKI	5 (18)	2 (7)
Baseline ANC		
Mean (SD)	5.1 (1.8)	4.1 (1.4)
Baseline ALC		
Mean (SD)	1.3 (0.6)	1.3 (0.5)
Baseline NLR		
Median (IQR)	3.9 (2.7–5.6)	3.0 (2.5–4.8)
ANC after 2 cycles		
Median (IQR)	3.7 (2.8–5.1)	4.5 (3.5–5.7)
ALC after 2 cycles		
Mean (SD)	1.4 (0.5)	1.2 (0.5)
NLR after 2 cycles		
Median (IQR)	2.9 (2.2–3.7)	3.9 (2.8–7)
